# Both Bupivacaine and Levobupivacaine inhibit colon cancer cell growth but not melanoma cells in vitro

**DOI:** 10.1007/s00540-018-2577-6

**Published:** 2018-11-13

**Authors:** Tianci Li, Lin Chen, Hailin Zhao, Lingzhi Wu, Joe Masters, Chongfang Han, Kazuyoshi Hirota, Daqing Ma

**Affiliations:** 1 Department of Anesthesiology, Shanxi Dayi Hospital, Shanxi Academy of Medical Sciences, Taiyuan, Shanxi China; 2 Anaesthetics, Pain Medicine and Intensive Care, Department of Surgery and Cancer, Faculty of Medicine, Imperial College London, Chelsea and Westminster Hospital, London, SW10 9NH UK; 3 Department of Anesthesiology, Hirosaki University Graduate School of Medicine, Hirosaki, Japan

**Keywords:** Bupivacaine, Levobupivacaine, Colon cancer, Melanoma

## Abstract

**Background:**

Retrospective studies indicate that the use of regional anaesthesia causes a reduction in cancer recurrence after oncological surgery, which could be due to anaesthetic’s negating effect on immunosuppression related to the surgical stress response. Local anaesthetics may also exert direct suppressive effects on malignant cells, an area where further investigation is urgently needed.

**Methods:**

Human colon cancer cells and human melanoma cells were cultured and then treated with 1 mM bupivacaine or levobupivacaine for up to 24 or 48 h. Their migratory ability was measured by scratch assay, proliferation determined with Ki67 immunofluorescence staining, and apoptosis accessed with annexin V and PI staining on flow cytometry. The effects of bupivacaine and levobupivacaine on cellular signaling and molecular response, specifically, on endoplasmic reticulum stress (ERS), were studied with immunostaining and western blot.

**Results:**

In colon cancer cells, treatment with bupivacaine and levobupivacaine significantly inhibited cell migration (**p < 0.01, ***p < 0.001; *n* = 4) and proliferation (**p < 0.01; *n* = 4), while increasing the expression of CHOP (***p < 0.001; *n* = 4) and decreased the expression of Grp78 (*p < 0.05; *n* = 4). These effects were not mirrored by melanoma cells, such that no significant increase in apoptosis was seen in either melanoma cell lines following treatment.

**Conclusion:**

These in vitro data suggested that both bupivacaine and levobupivacaine suppress colorectal adenocarcinoma cell proliferation and migration, which are concurrent with increased endoplasmic reticulum stress. Conversely, melanoma cells are more resilient to these two commonly used local anaesthetics. Further in vivo studies or clinical trials are needed.

## Introduction

Cancer is one of the leading causes of global mortality resulting in 8.2 million deaths worldwide in 2012, with 14 million newly diagnosed cases each year (expected to rise to 22 million annually in the next two decades) [1]. However, the specific mechanisms of tumourigenesis have not been completely uncovered, and despite significant advances in the development of targeted cancer therapies, excisional surgery supported by chemotherapy and/or radiotherapy remains the standard treatment for most patients with solid organ tumours. Accordingly, the impact of perioperative management (including anaesthetic drugs) on cancer prognosis following surgery has become an area of great interest [2–5]. Clinical retrospective data indicated that application of local anaesthetics during surgery is related to a lower level of cancer recurrence and metastasis [6, 7], and such observation prompts further investigation into the action of local anaesthetics on cancer biology.

Local anaesthetics are used in cancer surgery for local infiltration, peripheral nerve blockade, and central neuraxial anaesthesia as part of the patient’s perioperative analgesic regimen. Regional anaesthesia is often combined with general anaesthesia to reduce the quantity of opioids and volatile anaesthetic agents required [8]. In addition to this, the techniques of regional anaesthasia appear to have systematic anti-inflammatory effects that can inhibit the recurrence and metastasis of malignant cells by attenuating the immunosuppression associated with the surgical stress response. This immunosuppression inhibits the actions of immunocytes such as natural killer cells [9], which can kill residual tumour cells without deliberate activation, and hence undermines efforts to eradicate the malignancy. Study also indicates a direct inhibitory effect of amide local anaesthetic ropivacaine on cancer cells in vitro [10]. It has been demonstrated that blockade of voltage-gated sodium channels (a principal mechanism by which local anaesthetics block sensory nerve conduction) inhibits invasion of colon cancer cells. Amide local anaesthetics can also inhibit the metastasis of lung cancer cells by tumour necrosis factor-α-induced Src-activation and intercellular adhesion molecule-1 phosphorylation, both of which are independent of sodium channel blockade [11]. And recent work from our own group showed that bupivacaine induces apoptosis in ovarian and prostate cell lines [12]. Nevertheless, there is still the need for more research on the specific molecular mechanisms underlying these observations, and for translational evidences to pave ways for clinical randomized controlled trials.

Uncontrolled tumour growth leads to disturbance of the intracellular microenvironment, which significantly impairs the normal function of the endoplasmic reticulum (ER), the central organelle of the intracellular membrane system responsible for the synthesis, folding, modification, and transport of proteins. Dysfunction in proteostasis leads to accumulation of misfolded and unfolded polypeptides in the lumen of the ER, followed by relevant chaperone proteins activating further downstream pathways, which puts the cell under endoplasmic reticulum stress (ERS); this can lead to cell death if ERS goes unchecked. Cancer cells are especially susceptible to ERS because of uncontrolled protein synthesis, nutrient deprivation, and hypoxia following their rapid growth [13]. Further investigation into the role of ERS-related pathways in cancer pathophysiology and therapeutics is therefore warranted, and it has been demonstrated that the amide local anaesthetic lidocaine affects cancer cells through such pathways [14].

This study aims to explore the effects of two widely used amide local anaesthetics, bupivacaine and its S-enantiomer levobupivacaine, on colon cancer cells and melanoma cells. The expression of ERS-associated proteins, CHOP and Grp78, will be studied in treated groups to investigate whether these amide local anaesthetics exert their effects on cancer cells in part through ERS pathways.

## Materials and methods

### Cell culture

The human colorectal adenocarcinoma cell line Caco-2 and the human melanoma cell line A375 was purchased from Public Health England (Salisbury, UK). The culture medium used in this research was supplied by Gibco® RPMI 1640 (+ l-Glutamine, Life Technologies, Paisley, UK), 10% fetal bovine serum (FBS) (Fischer Scientific, Leicestershire, UK) and 1% Penicillin–Streptomycin (Sigma-Aldrich, Dorset, UK).

### Cell seeding in petri dishes

Cells prepared for experiments were cultured in 60 mm Petri dishes (Thermo Scientific) with density of 8 × 10^5^ cells/mL in 3 ml of medium per dish. Cell number was calculated with a hemocytometer (Hawksley, Lancing, USA) before seeding.

### Local anaesthetic treatment

Bupivacaine hydrochloride isotonic solution (Marcaine, 0.5%w/v, AstraZeneca, Luton, UK) and levobupivacaine hydrochloride isotonic solution (Chirocaine, 0.5%w/v, AstraZeneca, Luton, UK) were diluted with culture medium separately to reach the concentration of 1 mM, while Dulbecco’s phosphate buffered saline (DPBS/Modified 1×, Thermo Scientific, Utah, South Logan, USA) was added into culture medium at the same proportion as vehicle control. They were applied when cells grew to 90% confluence and their effects were determined with annexin V staining assay, immunostaining, and Western blotting.

### Scratch assay

The scratch assay was performed to assess the migration ability of cancer cells. After cells reach confluence in Petri dishes, horizontal and vertical cross lines were scratched on the cell monolayer by a p1000 plastic micropipette tip. Distance between the “cell-free” gap was almost identical. The previous culture medium was aspirated out and replaced with prepared medicine and control medium after gently washing the cell surface with culture medium to remove cellular debris. Snapping images of scratch area was taken with a microscope assisted with the digital camera (Olympus CK30, Tokyo, Japan) in the same frame and fields at 0 h, 24 h, and 48 h. Images were analysed by ImageJ software (National Institutes of Health, Bethesda, MD) to compare the mean percentage of closure area among groups.

### Flow cytometry

To assess the apoptosis of cancer cells, annexin V and propidium iodide (PI) staining assay was performed. After 24 h of treatment or control, adhesive cells were harvested through detachment by 0.25% trypsin and centrifugation at 1400 rcf for 10 min. After resuspending cells with 1 × Binding Buffer, 5 µl of fluorochrome-conjugated annexin V (Sigma-Aldrich, Saint Louis, USA) was added into 100 µl of cell suspension to stain intracellular phosphatidylserine (PS). Incubation was performed in dark under room temperature. 5 µl of propidium iodide staining solution (Sigma-Aldrich, Saint Louis, USA) was added into 100 µl of cell suspension. Flow cytometry (FACS Calibur, Becton Dickinson, Sunnyvale, CA, USA) was used to detect the percentage of apoptotic cells by FlowJo software (Treestar, Ashland, USA).

### Immunofluorescence

Cells treated with local anaesthetics for 24 h together with controls were fixed with 4% paraformaldehyde and blocked with 3% donkey serum [15] (Millipore, UK). Cells were incubated at 4 °C overnight in 3% DS 0.03% Triton-X phosphate buffered saline (PBST) with the following primary antibodies: anti-Grp78 (H-129) (1:200, rabbit polyclonal antibody; Santa Cruz Biotechnology, UK) to detect the general transducer of ERS Grp78, anti-GADD153 (F-168) (1:200, rabbit polyclonal antibody; Santa Cruz Biotechnology, UK) to detect the expression of CHOP, and anti-KI67 to visualize nuclear expression of Ki67 (1:200, monoclonal mouse antibody; DakoCytomation, Produktionsvej, Denmark) to determinate the proliferative state of cancer cells.

On the second day, cells were incubated in the corresponding secondary antibodies: fluorescein isothiocyanate (FITC)-conjugated donkey anti-rabbit IgG 1:200; Millipore, or FITC-conjugated donkey anti-mouse IgG (1:200; Millipore), and mounted with DAPI (Vector Laboratories). Cells were examined with a Nikon E1000M (Nikon, Surrey, UK) fluorescence microscope under 20× objective. Images were snapped with the identical exposure settings.

### Western Blot

Cells were lysed with cell lysis buffer (Cell-Signaling Technology), and the extracted protein fraction was quantified by Bradford protein assay (Bio-Rad laboratories, Hercules, CA, USA) to ensure equal loading of 50 µg of protein for each sample. Samples were loaded into NuPAGE® 4%-12% Bis-Tris Precast Gels (Thermo Scientific, UK) for electrophoresis. Protein bands were transferred from gel onto polyvinylidenedifluoride (PVDF) membranes, and incubated with primary antibodies-Grp78, GADD153 or cleaved caspase-3 p17 (H-60) (rabbit polyclonal antibody; Santa Cruz Biotechnology, UK) at dilution factor of 1:300. On the second day, membranes were incubated with secondary antibody (anti-rabbit HRP conjugated, Cell-Signaling Technology) for 1 h at room temperature at dilution factor of 1:1000, before development with enhanced chemiluminescence (ECL) system (Santa Cruz, Dallas, TX, USA). Protein bands were visualized by Syngene GeneSnap software (Syngene, Cambridge, UK). The intensity of grey scale of protein bands was assessed with ImageJ.

### Statistical analysis

Statistical analysis of all data was processed by GraphPad Prism version 5 (La Jolla, CA). The comparison between different groups was tested by one-way ANOVA and post-hoc Tukey test in Western blotting and immunofluorescence. Scratch assay was analysed by two-way ANOVA. Values were presented as mean ± Standard Deviation (SD), and a *p* value (two-tailed) < 0.05 was considered to be statistically significant.

## Results

### Bupivacaine and levobupivacaine inhibited the migration ability of Caco2 cells but not A375 cells

**Fig. 1:**
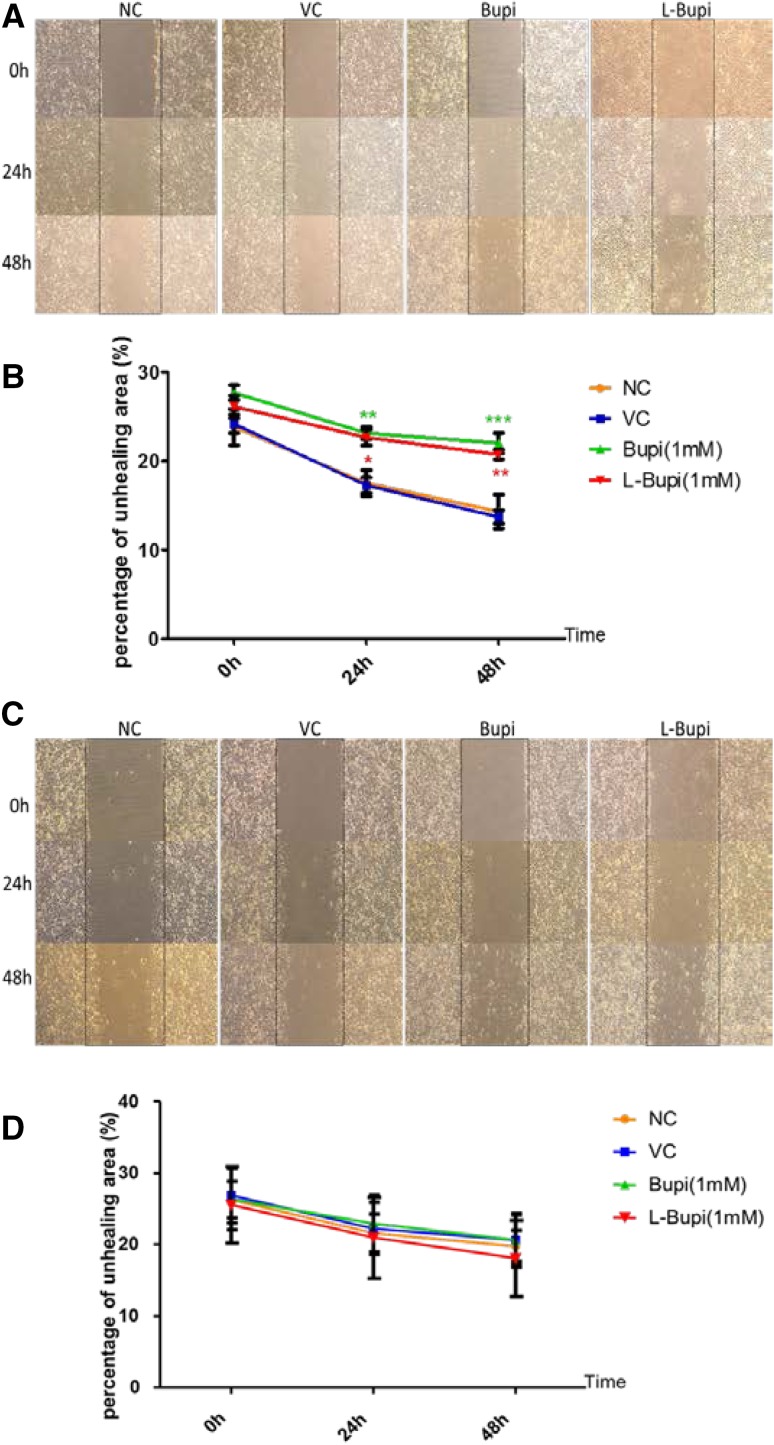
The effect of bupivacaine and levobupivacaine on migration ability of Caco2 cells and A375 cells. Representative microphotographs showing the scratch healing state after 24 h and 48 h of bupivacaine or levobupivacaine treatment in (**a**) caco-2 cells and A375 cells (**c**). Every image of scratch assay was taken under ×20 objective. **b, d** Illustrate the changes in percentage of unhealed area of caco-2 cells and A375 cells overtime. (data shown as mean ± SD; *n* = 4; *p < 0.05, **p < 0.01, ***p < 0.001; *NC* naïve control, *VC* vehicle control, *Bupi* application of 1 mM bupivacaine, *L-Bupi* application of 1 mM levobupivacaine)

As shown by the scratch assay, treatment with 1 mM bupivacaine or 1 mM levobupivacaine for 24 h and 48 h significantly decreased the gap closure rate of Caco2 cells (Fig. 1, b). Yet there was no significant difference in gap closure and migration ability following bupivacaine or levobupivacaine treatment in A375 cell line (Fig. 1c, d).

### Bupivacaine and levobupivacaine did not induce apoptosis in both cell lines but arrested the cell cycle of the Caco2 cell line

**Fig. 2:**
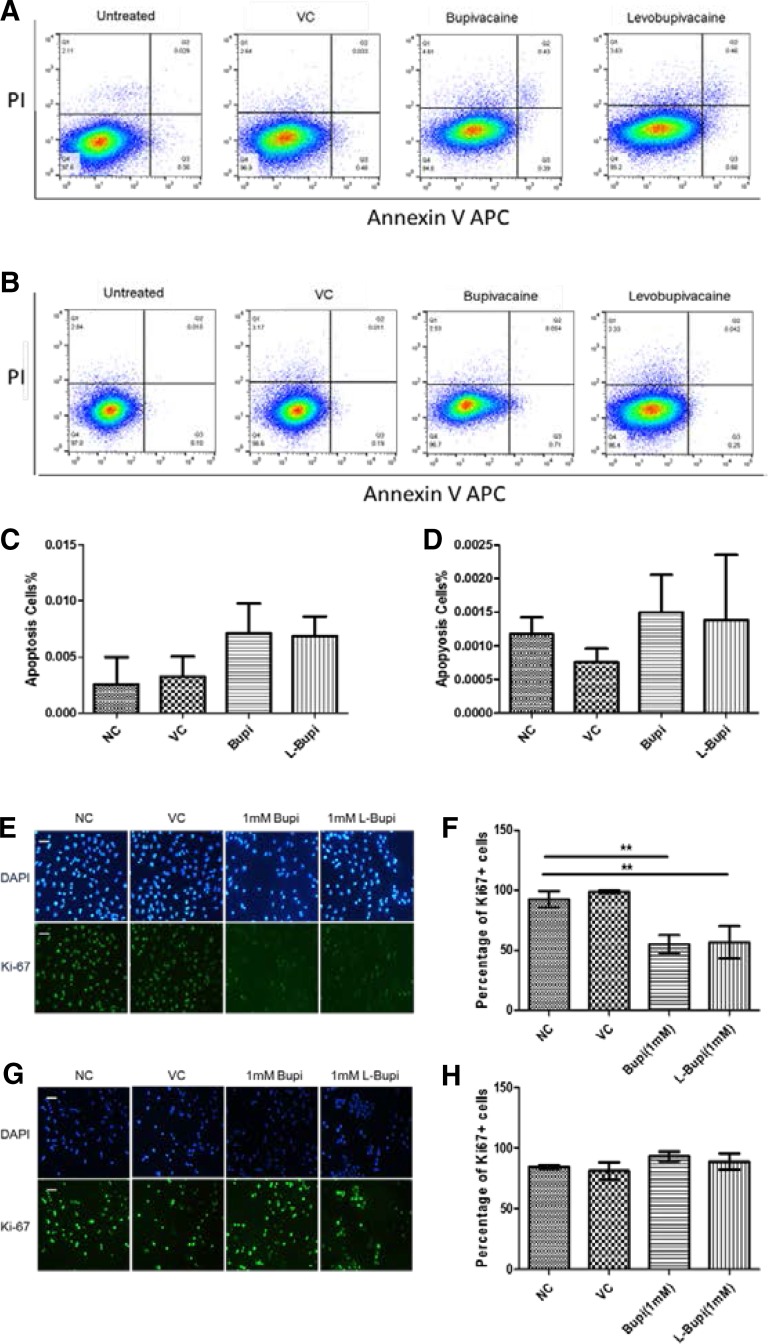
State of apoptosis and proliferation in Caco2 cells and A375 cells after treatment of bupivacaine and levobupivacaine. Each of the two cell lines was treated with 1 mM bupivacaine or levobupivacaine for 24 h. Cell distribution diagrams with PI and annexin V staining are shown for **a** Caco2 and **b** A375. Percentages of apoptotic Caco2 cells (**c**) and A375 cells (**d**) (*n* = 4). Representative immunofluorescence images of Ki-67 in Caco2 cells (**e**) and A375 cells (**g**) (×20 magnification; nuclei counterstained with DAPI; scale bar = 50 µm). Bar chart showing and comparing percentages of Ki67 + cells across different treatment groups in Caco2 cells (**f**) and A375 cells (**h**). (*n* = 3) (Results are mean ± SD; *p < 0.05; **p < 0.01; *NC* naïve control, *VC* vehicle control, *Bupi* 24 h treatment of 1 mM Bupivacaine, *L-Bupi* 24 h treatment of 1 mM Levobupivacaine)

Given that the application of the local anaesthetics affected cell healing, immunofluorescence staining was performed to evaluate tumour proliferation state. The mitosis marker, Ki-67 protein, which only exists in cells in the G1–M phases of cell cycle, but not in resting or damaged cells, was chosen as the proliferation marker. Bupivacaine and levobupivacaine significantly reduced the number of Caco-2 cells showing positive Ki67 nuclear staining, suggesting that both agents significantly inhibited cell proliferation in this cell line (Fig. 2e, f); on the other hand, both agents showed no significant effect on the nuclear level of Ki67 of A375 cells and their proliferation (Fig. 2g, h).

Annexin V and propidium iodide (PI) staining assays were performed to examine the apoptotic states of the Caco2 cells and A375 cells. Annexin V binds to phosphotidylserine (PS) when it translocates to the extracellular side of the cell membrane during the early stage of apoptosis. PI binds to DNA but is cell membrane-impermeable, such that it is excluded from viable cells until the late stages of apoptosis. The percentage of apoptotic cells in Caco2 cells and A375 cells remained at very low level (< 1%) following drug treatment and there was no significant difference across groups (Fig. 2c, d).

### Bupivacaine and levobupivacaine decreased the expression of Grp78 and increased the expression of CHOP in Caco2 cell line but not in A375 cell line

As the general transducer of ERS, Grp78 was detected by western blotting and immunofluorescence in the two cell lines after 24 h of treatment with 1 mM bupivacaine or 1 mM levobupivacaine.

**Fig. 3:**
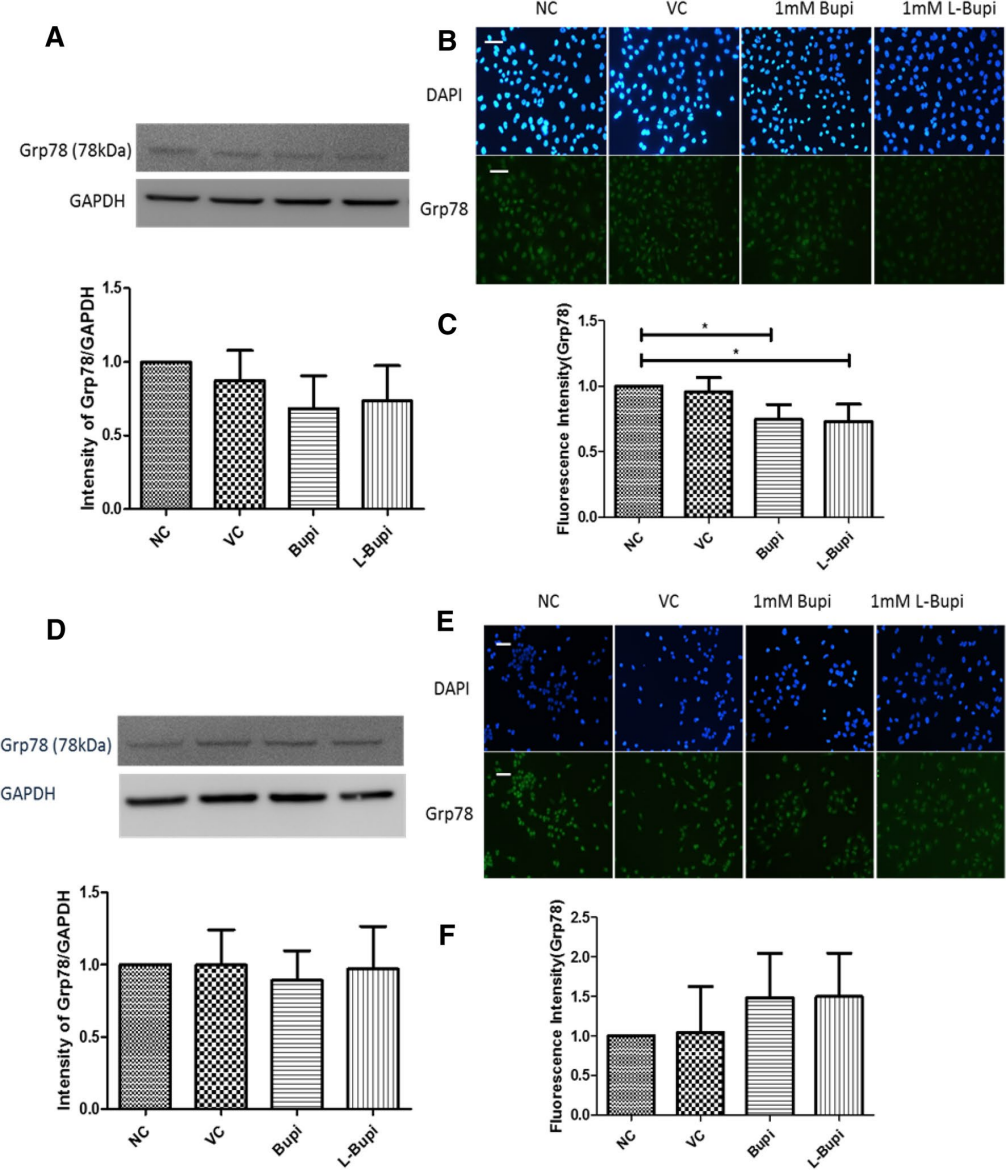
Expression of Grp78 after application of bupivacaine and levobupivacaine. Representative western blots and band density analysis of Grp78 in **a** Caco2 cells and **d** A375 cells. Grey scale of intensity values expressed as ratio relative to GAPDH. Representative immunofluorescence images of Grp78 in **b** Caco2 cells and **e** A375 cells (×20 magnification; nuclei counterstained with DAPI; scale bar = 50 µm). Immunofluorescence intensity of Grp78 in (C) Caco-2 cells and (F) A375 across different treatment groups (*n* = 4, data shown as mean ± SD; *p < 0.05; *NC* naïve control, *VC* vehicle control, *Bupi* 24 h treatment of 1 mM Bupivacaine, *L-Bupi* 24 h treatment of 1 mM Levobupivacaine)

In Caco2 cells, western blot testing showed no significant difference between all test groups (Fig. 3a), but immunofluorescent analysis demonstrated a reduction in Grp78 level in the bupivacaine or levobupivacaine treatment groups (*p* < 0.05; Fig. 3c). It is also evident from the immunofluorescence images that Grp78 was activated and localised into the nuclei, an observation that is consistent with its role as a heat-shock protein (Fig. 3b). In A375 cells, the expression of Grp78 was not significantly altered following drug treatment, when evaluated with either westernblot analysis or immunofluorescent staining(Fig. 3d–f).

**Fig. 4:**
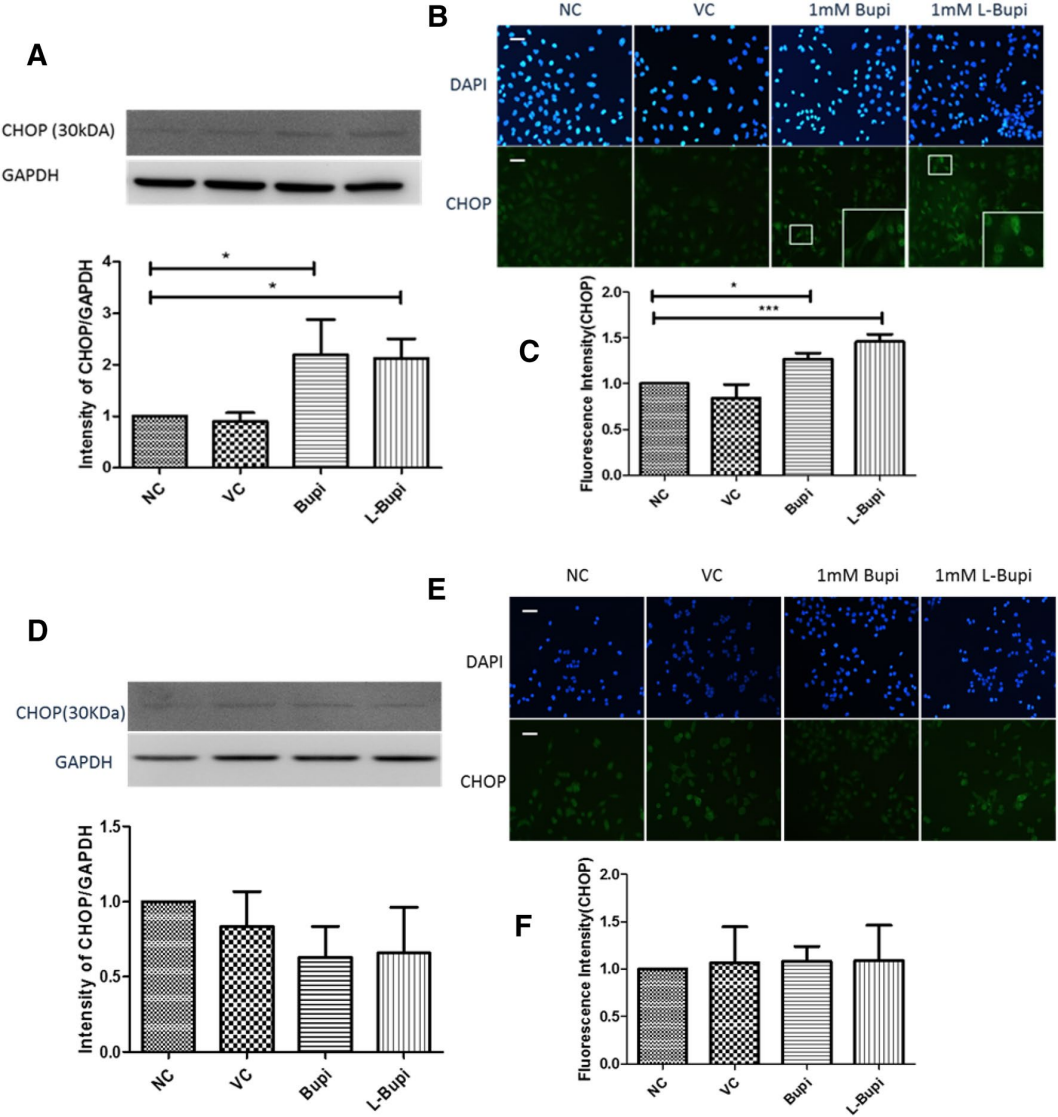
Expression of CHOP after application of bupivacaine and levobupivacaine. Representative western blots and band density analysis of CHOP in **a** Caco2 cells and **d** A375 cells. Grey scale of intensity values expressed as ratio relative to GAPDH. Representative immunofluorescence images of CHOP in **b** Caco-2 cells and **e** A375 cells (×20 magnification; nuclei counterstained with DAPI; scale bar = 50 µm). Immunofluorescence intensity of CHOP in **c** Caco-2 cells and **f** A375 cells across different treatment groups (*n* = 4, data shown as mean ± SD; *p < 0.05; *NC* naïve control, *VC* vehicle control, *Bupi* 24 h treatment of 1 mM Bupivacaine, *L-Bupi* 24 h treatment of 1 mM Levobupivacaine)

The application of 1 mM bupivacaine or 1 mM levobupivacaine for 24 h induced a significant increase in CHOP protein in Caco2 cells, as seen with both western blot analysis and immunofluorescence (*p* < 0.05; Fig. 4a–c). The immunofluorescent images demonstrated an increase in the nuclear localisation of CHOP after drug treatment (Fig. 4b). The A375 cell line showed no significant difference in CHOP expression after drug treatment (Fig. 4d–f).

## Discussion

Our results demonstrate that bupivacaine or levobupivacaine causes significant inhibition in cell migration ability and cell cycle arrest in the colorectal cancer Caco-2 cell line. Concurrent with such changes in cancer behavior are changes in the expression of the ERS proteins, to suggest that the anti-migratory and anti-proliferative effects of local anaesthetics on colon cancer cell may be mediated through endoplasmic reticulum stress; and the lack of ERS response in melanoma cells following local anaesthetics treatment may also explain their unchanged migration and proliferation behaviors.

In this study, after treatment of 1 mM bupivacaine and levobupivacaine for 24 h, the percentage of Ki67 + cells significantly decreased in the Caco-2 cell, without significant increase in apoptosis. Previous studies have also indicated that both bupivacaine and levobupivacaine can inhibit the proliferation of cancer cells without inducing cell death [16].

Heat shock proteins exist in the lumen of endoplasmic reticulum and many members of this family act as chaperones to facilitate protein folding. Of this group, the 78 kDa glucose-regulated protein (Grp78) is one of the most important regulators of ERS. Grp78 combines with hydrophobic residues of immature proteins for subsequent processing. When under normal condition, Grp78 can deactivate three important sensors of ERS—PKR-like endoplasmic reticulum kinase (PERK), inositol-requiring enzyme 1α (IRE 1α), and activation transcription factor 6 (ATF6). However, with ER stress, increased levels of unfolded polypeptides in the ER leads to excessive occupation and sequestration of Grp78, which becomes dissociated from these three transducers [17]. Subsequently, the dissociation of Grp78 from PERK, IRE1, and ATF6 will “dis-inhibit” the downstream ERS signaling pathways. This ERS response, also known as the unfolded protein response (UPR) [18], is a homeostatic mechanism that can exert either a cytoprotective effect, or induce apoptosis depending on the severity and duration of stress.

In cancer cells, it is possible that activation of ERS mechanisms could promote cell survival, at least during the initial phase. Following dissociation of Grp78, PERK-eIF2α (phosphorylate eukaryotic initiation factor2α) axis is activated and suppresses polypeptide synthesis through translational attenuation to prevent further accumulation of misfolded proteins within the ER, and thus relieving the cancer cells from intracellular stress to favor cancer survival [19]. Likewise, the activation of other ERS signal transducers, IRE1 and ATF6, can also favor tumour growth and enhance cell tolerance to stress by activating the mRNA of transcription factor X-box-binding protein 1 (XBP-1) and upregulating expression of major ER chaperones (including Grp78) and ERAD components [20, 21].

On the other hand, with ongoing disruption of ER homeostasis, the aforementioned UPR mechanisms will start to downregulate efforts in resisting ERS, while strengthening their tendency to induce cell cycle arrest or programmed cell death [22]. The PERK-eIF2α pathway is the leading promoter of the transcription factor C/EBP homologous protein (CHOP) [23], which upregulates a range of proteins that drive apoptosis (including growth-arrest and DNA-damage inducible protein 34 [GADD34], ER oxidase 1α [ERO1α], BCL2-like 11, tribbles-related protein 3 [TRB3], and death receptor 5 [DR5]) and downregulates anti-apoptotic factors (such as BCL-2) [24, 25]. In view of the above, our collective findings that treatment of Caco2 cells with bupivacaine and levobupivacaine is associated with a decrease in the expression of Grp78 and an increase in the expression of CHOP suggests that these amide local anaesthetics can enhance ERS. Such enhancement in ERS may contribute to local anaesthetics’ inhibitory effect on cell migration and proliferation. Interestingly, in some retrospective clinical studies, Grp78 and CHOP seem to play opposing roles in ERS in informing the prognosis of cancer patients. Matsuo et al. collected samples of both uterus and visceral adipose tissue from endometrial cancer patients and found that a more advanced stage and deeper invasion were associated with higher expression of Grp78 [26]. Another study demonstrated that in breast cancer patients, high expression of CHOP correlated with longer survival and a lower recurrence rate [27].

Furthermore, it is worth mentioning that local anaesthetics have wide range of uses in clinical practice and their plasma concentrations can vary widely. In this regard, although the concentration of bupivacaine and levobupivacaine used in the present study (1 mM) far exceeds the plasma concentrations of bupivacaine and levobupivacaine in abdominal wall block, with mean peak plasma concentrations ranging between 1 and 3µM [28, 29], local anaesthetics are also administered to patients through intraperitoneal instillation during surgery and/or for post-operative pain relief, and it was found such administration can reduce systemic cytokine and cortisol levels [30, 31]. One can argue that different routes of administration makes it highly plausible for the local anaesthetics to reach tumour removal sites at greater concentrations, for example, after colorectal cancer excision, direct infiltration of local anaesthetics are used clinically and its concentration can reach a considerable level locally, and even higher when administered topically. Nevertheless, as the nature of a proof of concept study of its kind, our data showed that the two local anaesthetics tested in our study did not enhance cancer malignancy and the implication is that they may be favorable for cancer patient use during anaesthesia for surgery. Taken together, one may speculate that the local anaesthetics may prevent local cancer recurrence by suppressing proliferation of cancer cells that have been mobilized and scattered during tumour resection; nonetheless, further time- and concentration-dependent studies are needed to support such hypothesis.

In conclusion, we have demonstrated a direct inhibitory effect of bupivacaine and levobupivacaine on colon cancer cell migration and proliferation, which are associated with increased endoplasmic reticulum stress. In contrast, these observations were not made in the melanoma cell line studied. These findings may indicate elevated ERS as a candidate mechanism for anaesthetics to exert anti-tumour effect; however, further mechanistic studies are warranted before concluding such causal relationship. Ultimately, this work could lead to clinical studies examining the “anti-cancer” characteristics of amide local anaesthetics to further benefit cancer patient care.
